# Identification of differentially expressed genes involved in spore germination of *Penicillium expansum* by comparative transcriptome and proteome approaches

**DOI:** 10.1002/mbo3.562

**Published:** 2017-12-05

**Authors:** Ting Zhou, Xiaohong Wang, Jin Luo, Bishun Ye, Yingying Zhou, Liwan Zhou, Tongfei Lai

**Affiliations:** ^1^ Key Laboratory for Quality and Safety of Agricultural Products of Hangzhou City College of Life and Environmental Sciences Hangzhou Normal University Hangzhou China; ^2^ Research Centre for Plant RNA Signaling College of Life and Environmental Sciences Hangzhou Normal University Hangzhou China

**Keywords:** germination, iTRAQ, *Penicillium expansum*, RNA sequencing

## Abstract

In this study, *Penicillium expansum*, a common destructive phytopathogen and patulin producer was isolated from naturally infected apple fruits and identified by morphological observation and rDNA‐internal transcribed spacer analysis. Subsequently, a global view of the transcriptome and proteome alteration of *P. expansum* spores during germination was evaluated by RNA‐seq (RNA sequencing) and iTRAQ (isobaric tags for relative and absolute quantitation) approaches. A total of 3,026 differentially expressed genes (DEGs), 77 differentially expressed predicted transcription factors and 489 differentially expressed proteins (DEPs) were identified. The next step involved screening out 130 overlapped candidates through correlation analysis between the RNA‐seq and iTRAQ datasets. Part of them showed a different expression trend in the mRNA and protein levels, and most of them were involved in metabolism and genetic information processing. These results not only highlighted a set of genes and proteins that were important in deciphering the molecular processes of *P. expansum* germination but also laid the foundation to develop effective control methods and adequate environmental conditions.

## 1 INTRODUCTION

*Penicillium expansum*, one of major causal agents of blue mold rot, is a commonly distributed and destructive phytopathogen. Besides its usual hosts of deciduous fruits such as apples, pears, and peaches and products derived from them, it can infect some less common hosts including vegetables, kiwifruits, tomatoes, strawberries, and cherries (Morales, Marín, Ramos, & Sanchis, 2010; Zhu, Yu, Brecht, Jiang, & Zheng, 2016) and lead to extensive maceration utilizing a common mechanism of tissue acidification (Barad et al., 2012). In addition, *P. expansum* is an important producer of the mycotoxin patulin (Puel, Galtier, & Oswald, 2010). As a nonvolatile secondary metabolite, patulin produces acute and chronic toxicity, primarily genotoxicity and immunotoxicity, as well as cytotoxicity in humans. Ingesting patulin can lead to nausea, vomiting, gastrointestinal trauma, kidney damage, the formation of cancerous tumors, genetic mutations, and embryonic developmental defects (Barad, Sionov, & Prusky, 2016; Glaser & Stopper, 2012). The assessment of the health risks due to patulin consumption by humans resulted in the US Food and Drug Administration (FDA) and the Commission for the European Community (EC) setting a maximum tolerance limit of 50 μg·L^−1^ in apple‐derived food products. EC Regulation 1881/2006 set the maximum limit of patulin for certain contaminants in food equal to 10 μg·kg^−1^ (or 10 μg·L^−1^) in apple juice and solid apple products for infants and young children (Sarubbi, Formisano, Auriemma, Arrichiello, & Palomba, 2016; Snini et al., 2016). In addition, *P. expansum* is a psychrophilic mold with a minimum growth temperature near freezing and an optimum growth temperature of 25°C. It can be easily disseminated by different vectors in orchards and by equipment (Morales et al., 2010). Therefore, *P. expansum* may cause serious economic losses to the international horticultural industry every year.

In addition to proper sanitation and careful handling during storage and processing, cold temperature, fungicide, controlled atmosphere, natural antimicrobials from plants, generally recognized as safe substances, and antagonistic microorganisms are usually utilized to inhibit the growth and patulin production of *P. expansum* (Cheon et al., 2016; Daniel, Lennox, & Vries, 2015; De Paiva et al., 2017; Moake, Padilla‐Zakour, & Worobo, 2005; Morales et al., 2010). However, the overuse of synthetic fungicides has resulted in extensive concern about its effects on the environment and human health. In addition, the overuse of certain control strategies has led to increasing resistance of pathogens (Palou, Ali, Fallik, & Romanazzi, 2016). Therefore, it is imperative to understand the genetic basis of the infection, pathogenicity, toxigenicity, and virulence before developing new ecologically friendly control approaches and risk assessment models.

Fungal asexual spores that involve reproduction without the fusing of gametes are the main form of contamination or inoculation, since they are easily dispersed through the air or water. These spores are considered resistant to a broad spectrum of unfavorable environmental conditions due to both their cell wall properties and the accumulation of protective compatible solutes in the cytoplasm (Latgé & Beauvais, 2014). Mature conidia possess a condensed cell wall and a dense cytoplasm that contains a high content of energy‐storage materials such as the major carbon storage compounds trehalose and mannitol and an exogenously imposed dormancy (Voegele et al., 2005). Germination of the fungal conidia refers to the emergence of cells from resting asexual spores and that form sporeling hyphae or thalli under optimal growth conditions (Van Leeuwen et al., 2013). During this process, the hydrophobicity of the conidia results a progressive reduction that leads to the swelling of the conidia knows as isotropic growth and subsequent polarized growth characterized by germ tube formation (Dague, Alsteen, Latgé, & Dufrêne, 2008). It greatly affects fungal aggressiveness and colonization and can be considered to be one of the main steps of the disease cycle. Various studies have been conducted on the physiological and biochemical changes of *P. expansum* spores under different environment conditions including pH, nutrients, antimicrobial substances, temperature, and atmosphere composition (Kalai, Anzala, Bensoussan, & Dantigny, 2017; Long et al., 2017; Nanguy, Perrier‐Cornet, Bensoussan, & Dantigny, 2010). Ballester et al. (2015) and Li et al. (2015) have completed genomic sequencing of *P. expansum* and some other *Penicillia* strains that will aid further molecular genetic analysis. However, no information is available regarding the intrinsic flexibility and variation in *P. expansum* at the transcriptional or translational level at different developmental stages such as germination.

Therefore, this study aimed to assess the transcriptome and proteome alternation of *P. expansum* during its germination stage using high throughput sequencing techniques. The results should provide useful information to better understand the molecular basis of development and pathogenicity of *P. expansum* and help to develop efficient control strategies.

## 2 MATERIALS AND METHODS

### 2.1 Fungal isolation and identification

All of the fruits at commercial maturity were bought from a local market in the Xiasha District, Hang Zhou, China. The fungal pathogen *P. expansum* was initially isolated from a naturally infected apple (*Malus domestica* Borkh cv. Red Fuji) fruit with typical blue mold symptoms and maintained on potato dextrose agar (PDA) plates at 25°C. The pathogen was re‐inoculated on apple, orange (*Citrus reticulata* Blanco.), jujube (*Ziziphus jujube* Mill. cv Dongzao), tomato (*Lycopersicon esculentum* Mill.), and pear (*Pyrus bretschneideri* Rehd.) fruits to observe its morphological characteristics. Total genomic DNA of the pathogen was extracted using a DNeasy Plant Mini Kit (Qiagen, Germany) following the manufacturer's instructions. The rDNA‐ITS was amplified using the universal primers ITS4 (TCCTCCGCTTATTGATATGC) and ITS5 (GGAAGGTAAAAGTCGTAACAAG). The amplified products were purified by the Roche kit and sequenced by Shanghai Sunny Biotechnology Co., Ltd. The sequencing results were analyzed in http://blast.ncbi.nlm.nih.gov/blast.cgi. The distant tree of the blast results was produced by BLAST pairwise alignments, and the parameters were as follows: Tree method: Neighbor Joining; Max Seq Difference: 0.75; Sequence Label: Taxonomic Name.

### 2.2 Fungal physiological measurements

Conidial suspensions were obtained by flooding the sporulating cultures of *P. expansum* with sterile distilled water containing 0.05% (v/v) Tween‐20. A suitable aliquot of fresh spore suspension was added to a 250‐ml conical flask containing 100 ml potato dextrose broth (PDB) to obtain a final concentration of 1.0 × 10^6^ spores·ml^−1^ with the aid of a hemocytometer. The germination rate and germ tube length of the spores were microscopically determined during an incubation of period of 3–16 hr at 25°C on a rotary shaker at 200 rpm. 4′, 6′‐diamidino‐2‐phenylindole (DAPI) was utilized at a concentration of 50 mg/L to facilitate observation of the morphology of fungal spores as described by Lai et al. (2015). The dry weight of the hyphae was measured after centrifugation, and then they were repeatedly washed with distilled water and dried at 60°C in an oven to a constant weight at the indicated time. The mycelial growth in vitro and in vivo was measured daily on PDA plates and apple fruits separately as described by Lai et al. (2017).

### 2.3 Determination of patulin production

Fungal spores with a final concentration of 1.0 × 10^7^ spores/ml were cultured in PDB medium under static conditions at 25°C for 2, 3 and 4 days. After centrifugation, the supernatant was filtered through a 0.22 μm syringe filter (Albet, Spain), and then 10 μl of the filtrates was injected into a reversed‐phase HPLC (high performance liquid chromatography) system with a Waters XTerra RP18 column (Waters, USA) for the quantitative determination of the patulin concentration. The mobile phase consisted of 95% water and 5% acetonitrile. The operational parameters were as follows: a flow rate of 0.8 ml/min, a column oven temperature of 18°C, and a detector wave length of 276 nm.

### 2.4 RNA‐Seq analysis

Fungal spores were cultured in PDB medium under shaking conditions for 6 and 12 hr as described previously. The harvested spores and mycelia were washed twice by sterile distilled water and quickly frozen with liquid nitrogen for subsequent RNA‐Seq and iTRAQ analysis. Total RNA preparation and sequencing were performed using a service from Beijing Genomics Institute (BGI) Co., Ltd. The experiment was repeated once. Briefly, high‐quality total RNA with a 28S:18S ratio >1.5 and an absorbance 260/280 ratio between 1.7 and 2.0 was extracted using TRIzol reagent (Invitrogen, USA) following the manufacturer's instruction. The cDNA library for each sample was constructed utilizing TruSeq RNA Sample Preparation Kits v2 (Illumina, USA) according to the manufacturer's instructions. After quantification and qualification during the quality control step, the sample library was sequenced on the BGISEQ‐500 platform.

The raw data were filtered to remove adaptors and reads in which unknown bases were more than 10% or the percentage of low quality bases was over 50%. The remaining clean reads were stored as FASTQ format and mapped to reference (https://www.ncbi.nlm.nih.gov/Taxonomy/Browser/wwwtax.cgi?id=27334) utilizing *Bowtie2* (http://computing.bio.cam.ac.uk/local/doc/Bowtie2.html#) and *HISAT* (http://ccb.jhu.edu/software/hisat2/manual.shtml). The gene expression level was quantified utilizing the software package RSEM (Li & Dewey, 2011). Then, samples were first grouped so that the comparison between every two groups as a pairwise of different developmental stages was performed later. Differentially expressed gene (DEG) screening utilized the NOISeq method (Tarazona, Carcía‐Alcalde, Dopazo, Ferrer, & Conesa, 2011) according to the following criteria: fold change ≥ 2 and diverge probability ≥ .8. The detailed data are provided in Table S1. Gene ontology annotation and enrichment analysis of the DEGs were performed using WEGO software based on the Gene Ontology Consortium (http://www.geneontology.org/). The Kyoto Encyclopedia of Genes and Genomes (KEGG) database (http://www.kegg.jp/kegg/pathway.html) was used to conduct pathway enrichment analysis of the DEGs. The normalized data table is provided in the appendices in Table S1. The hmmsearch were used to search the characteristics of the domains in the PlantTFDB database (http://plntfdb.bio.uni-potsdam.de/v3.0/) and then to conversely predict if a gene codes for a transcription factor. Transcription factors related to DEGs are listed in Table S2. The clean reads could be acquired in Sequence Read Archive (SRA) (https://trace.ncbi.nlm.nih.gov/Traces/sra/). The accession IDs for Pe‐6h‐Replicate1, Pe‐6h‐Replicate2, Pe‐12h‐Replicate1 and Pe‐12h‐Replicate2 are SRR5830890, SRR5839793, SRR5819793 and SRR5816892, respectively.

### 2.5 iTRAQ‐based quantitative proteomics analysis

Isobaric tags for relative and absolute quantitation (iTRAQ) analysis was performed utilizing a service from LC‐Bio of LC Science (USA) as described by Lai et al. (2016). Protein preparation utilized the standard extraction procedure of LC‐Bio of LC Science. One hundred micrograms of the total protein was digested with Trypsin Gold (Promega, USA) at a ratio of 30:1 (protein: trypsin) at 37°C for 16 hr. The peptides were reconstituted in 0.5 mol·L^−1^ TEAB and processed according to the manufacturer's instructions for the 8‐plex iTRAQ reagent (Applied Biosystems, USA). The labeled peptide mixtures were loaded onto an LC‐20AB HPLC Pump system (Shimadzu, Japan) with a 4.6 × 250 mm Ultremex SCX column and a Strata X C18 column (Phenomenex). After SCX chromatography, LC‐ESI‐MS/MS analysis was performed utilizing an LC‐20AD NanoHPLC (Shimadzu, Japan), a TripleTOF 5600 System fitted with a NanoSpray III source (AB SCIEX, USA) and a pulled quartz tip as the emitter (New Objective, USA).

Raw data files acquired from the Orbitrap were converted into MGF files utilizing Proteome Discoverer 1.2 (Thermo, USA) and the MGF files were searched. Protein identification was carried out utilizing the Mascot search engine (Matric Science, UK). A mass tolerance of 0.05 Da for intact peptide masses, 0.1 Da for fragmented ions, and one missed cleavage in the trypsin digests were permitted. The parameters of the mass values [monoisotopic], variable medications [Gln‐ > pyro‐Glu (N‐term Q), Oxidation (M), iTRAQ8plex (Y)], instrument type [Default], and fixed medications [carbamidomethyl (C), iTRAQ8plex (N‐term), iTRAQ8plex (K)] were also set. The quantitative protein ratios were weighted and normalized utilizing the median ratio in Mascot. The proteins whose ratio with *p*‐value < .05 and fold change > 1.2 in two biological repeats were considered to be the differentially expressed proteins (DEPs). Functional annotations of the proteins were conducted using the Blast2GO program against the non‐redundant protein database (NR; NCBI). The Kyoto Encyclopedia of Genes and Genomes (KEGG) database (http://www.kegg.jp/kegg/pathway.html) and the COG database (http://www.ncbi.nlm.mih.gov/COG) were used to classify and group these identified proteins. The statistics of GO and pathway enrichment of DEPs are shown by ggplot2 (http://ggplot2.org/) in Figure 4. The detailed data are shown in Table S3.

Comparison of the RNA‐seq and iTRAQ datasets was based on the semantic similarity of their GO terms. Detail information of the common DEGs acquired from the two approaches is presented in Figure 6. HemI1.0.1 was utilized to prepare heat maps (Deng, Wang, Liu, Cheng, & Xue, 2014).

### 2.6 Statistical analysis

Except for the experiments where specified, the data were pooled across three independent repeat experiments, and statistical analysis were performed with SPSS software (SPSS Inc., Chicago, IL, USA). Analysis of variance (ANOVA) was used to compare more than two means, and Duncan's multiple range test was used for mean separations. Differences at *p *<* *.05 were considered to be significant.

## 3 RESULTS

### 3.1 Identification of a strain of *P expansum* from decaying market fruit in the Chinese province of Zhejiang

**Figure 1:**
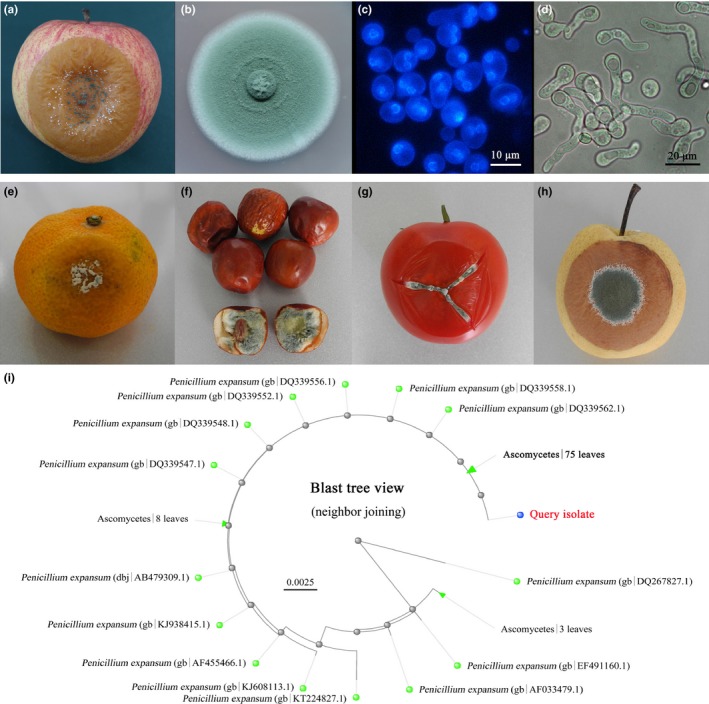
Genetic and morphological features of blue mold isolated from apple fruits. Symptoms of blue mold on apple (a), orange (e), jujube (f), tomato (g) and pear (h) fruits are shown. Colonial morphology of *Penicillium expansum* on PDA medium (b), morphology of spores stained by DAPI after 6 hr of culturing (c), and spore formation after 12 hr of culturing in PDB medium (d) are illustrated. The distant tree of blast results produced by BLAST pairwise alignments demonstrated the fungal genetic background (i)

The fungus, isolated from decaying market fruit, proved to be a major causative agent by artificial infection tests, and it was highly pathogenic. Therefore, we decided to identify and characterize this pathogen by morphological observation and rDNA‐internal transcribed spacer analysis. After an artificial re‐inoculation in vivo and in vitro, the cultural and morphological characteristics of the purified pathogen were consistent with those of blue mold. In apple fruits, the color of the lesions was light brown. The infected tissue with an earthy‐musty odor and blue pustules became soft and mushy, and had a watery consistency. On the older lesions, the pathogen formed typically brush‐like sporulating structures that produced single celled spores (Figure 1a). The spore masses initially appeared as white mycelia that then turned bluish‐green in color from the edge to the center of the colonies on PDA plates (Figure 1b). The conidia were spherical or elliptical and contained one or multiple nuclei (Figure 1c and d).

The pathogen was also highly pathogenic to orange, jujube, tomato, and pear fruits (Figure 1e–h). After amplification using ITS universal primers, an approximately 800‐bp fragment was identified (Figure S1). Through sequencing and MegaBLAST analysis, the submitted sequence was found to match perfectly with the ITS of *P. expansum* strains (Figure 1i). Therefore, the isolated pathogen was confirmed as *P. expansum*.

### 3.2 Growth dynamic analysis of *P. expansum* in vitro and in vivo

**Figure 2:**
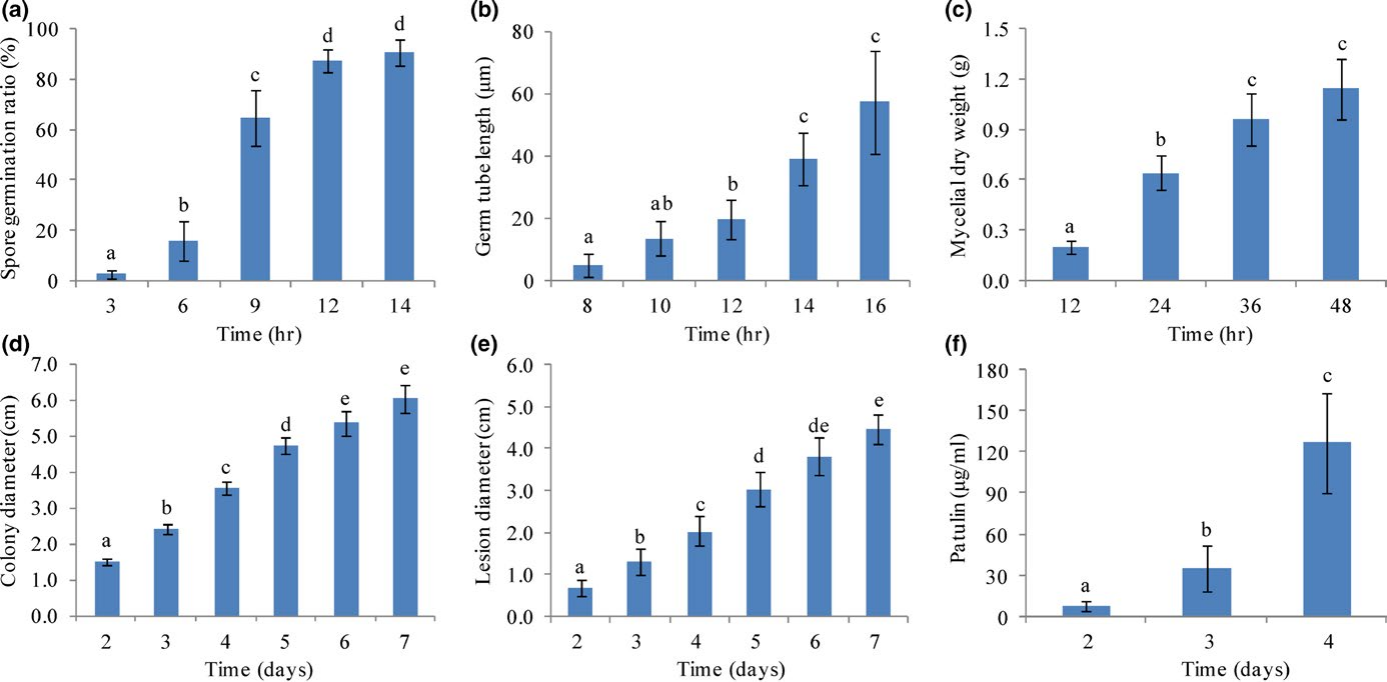
Growth dynamic analysis of *P. expansum* in vitro and in vivo. Spore germination (a), germ tube length (b), mycelial dry weight (c), colony diameter on PDA (d), and lesion diameter on apple fruits (e) of *P. expansum* were determined at the time points indicated. Patulin production of *P*. *expansum* under static conditions was detected by a reverse‐phase HPLC system as well (f). Bars represent the standard deviation of the means of three independent experiments. Lower case letters indicate significant differences at *p *<* *.05 at each time point

As one of the most prevalent postharvest diseases, the growth dynamics of *P. expansum* that partially represent fungal competitiveness and dominance are important to determine. In this study, several growth indicators were determined at different incubation times. After 6 hr of cultivation in PDB, the average volume of the spores was visually enlarged. After 12 hr, the germination rate had reached more than 90% (Figure 2a). After 16 hr, the average length of the germ tube was approximately 60 μm (Figure 2b). After 48 hr, the mycelial dry weight derived from 1.0 × 10^8^ spores had reached about 1.2 g (Figure 2c). After 6 days of cultivation on PDA, colonies nearly extended to the entire petri dish (Figure 2d). In addition, *P. expansum* was strongly pathogenic to apple fruits and was highly destructive (Figure 2e). After 6 days of infection, the lesion diameter on apple fruits was almost 4 cm. Patulin is an important indicator of pathogenicity of *P. expansum*. Patulin production significantly increased with the time of culture. The concentration of patulin was greater than 130 μg·ml^−1^ after 4 days of incubation (Figure 2f).

### 3.3 Identification and functional classification of DEGs during spore germination of *P. expansum*

**Figure 3:**
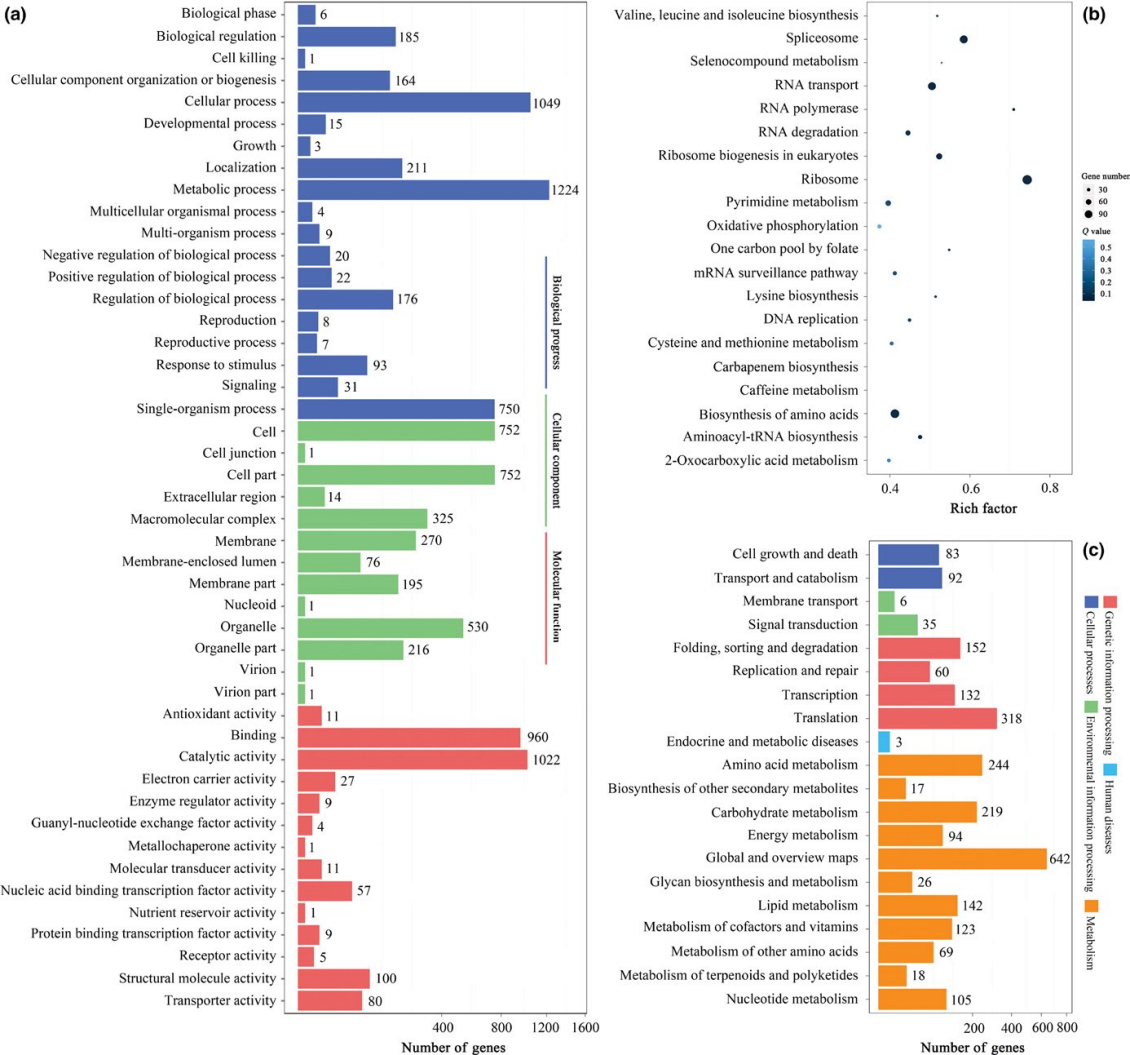
GO function classification, pathway enrichment statistics and KEGG classification of DEGs. (a) GO functional classification of DEGs for each pairwise. The *X* axis indicates the number of DEGs (the number is presented by its square root value). The *Y* axis represents GO terms. All of the GO terms are grouped into three ontologies: blue is for biological process, brown is for cellular component and orange is for molecular function; (b) Statistics of the pathway enrichment of DEGs in each pairwise. The rich factor is the ratio of DEGs numbers to all gene numbers annotated in this pathway term. A greater rich factor indicates greater intensity. The *Q* value is the corrected *p* value ranging from 0 to 1, and a lower *Q* value indicates greater intensity. The top 20 of the enriched pathway terms are displayed. (c) KEGG classification of DEGs. The *X* axis indicates the number of DEGs. The *Y* axis represents the second KEGG pathway terms. All of the second pathway terms are grouped in the top pathway terms indicated in different colors. The detailed information of these DEGs is listed in Table S1

RNA‐seq‐based transcriptome analysis has several advantages such as the production of large‐scale data, high reproducibility, enhanced sensitivity, and relatively low cost. Thus, it serves an essential role to identify genetic networks underlying cellular, physiological, biochemical and biological systems. To explore and determine the underlying molecular and cellular processes during spore germination of *P. expansum*, two key time points of 6 hr and 12 hr were selected for transcriptome profiling based on light microscopy studies. After 6 hr, significant multiple developmental stages were observed, and the dormant spores had been fully activated and were initiating vegetative growth with cell wall expansion. After 12 hr of incubation, the spores had almost finished germination and generated germ tubes that would grow and develop into hyphae. Our goal was to focus on identifying genes with stage‐specific expression patterns or altered expression levels corresponding to substantial morphological and physiological changes between the two important stages. Finally, about 23.96 million raw sequencing reads and about 23.95 clean reads of each sample were generated after filtering out the low‐quality ones. The average mapping ratio with the reference gene is 76.13%, and the average genome mapping ratio is 97.51%. A total of 3,026 genes were found to have changed abundantly during spore germination of *P. expansum*. Compared with those after 6 hr of incubation, the expression of 1,814 DEGs was down‐regulated, while 1,212 DEGs were up‐regulated. Detailed information on all of the DEGs is summarized in Table S1. The biological functions and intracellular distribution of these DEGs are shown in Figure 3a. The majority of these DEGs possessed binding capability or catalytic activity and were involved in cellular, metabolic and single‐organism processes. The pathway enrichment statistics and KEGG classification of the DEGs are shown in Figure 3b and c. The pathway classification indicates that most of the DEGs participate in metabolism and genetic information processing. In addition, to investigate whether master regulators control the transcriptional change during spore germination, a total of 77 transcription factors among DEGs belonging to the Alfin‐like, bHLH BSD, bZIP, C2C2‐GATA, C2H2, C3H, FHA, HSF, MADS, MYB‐related, TIG, and Zn‐clus families are predicted (Table S2). However, the functions of most of them are unknown.

### 3.4 Identification and functional classification of DEPs during spore germination of *P. expansum*

**Figure 4:**
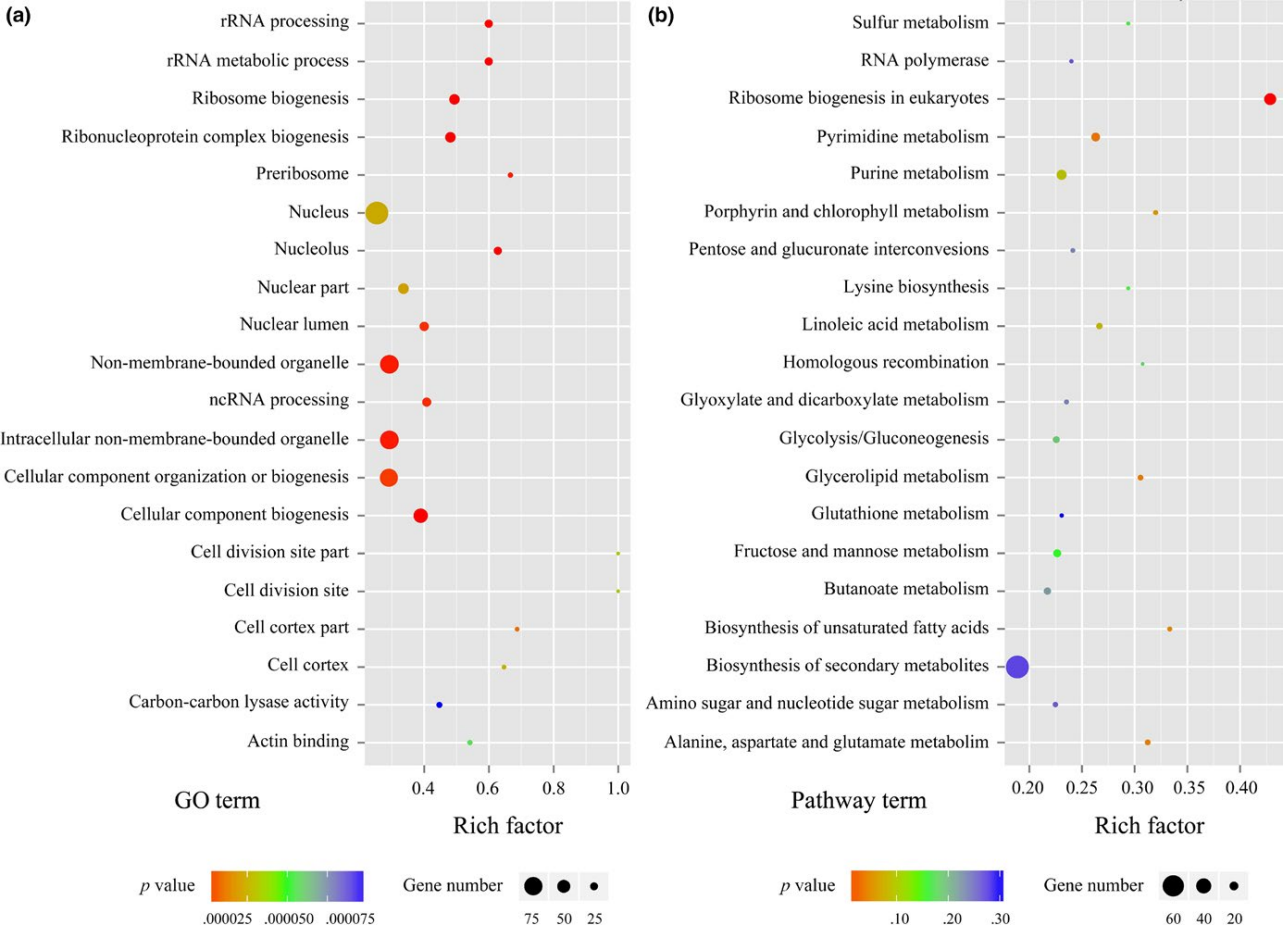
Scatter plots of the top 20 Gene Ontology (a) and KEGG (b) enrichment of DEPs involved in spore germination of *P*. *expansum*. A deeper color in the color code represents the higher confidence for the biological process. Rich factor: the number of DEPs in one GO or KEGG/the number of total identified proteins in the same GO or KEGG. A higher value indicated higher enrichment level. The detailed information of these DEPs is listed in Table S3

Although transcriptome profiling provides substantial biological information, its ability to provide a comprehensive perspective for this biological target is limited. mRNA profiling cannot capture the regulatory processes or post‐transcriptional modifications that might affect the amount of active proteins. As a complement to genome and transcriptome analysis, proteomic analysis has proven to be a powerful method to offer a more direct analysis of cellular events and facilitate drawing accurate functional inferences from gene expression data. Thus, an iTRAQ‐based quantitative proteomic analysis was utilized to gain a global view of proteome alterations of *P. expansum* spores during germination. Two independent biological replicates for each sample were used for labeling. A total of 3,495 proteins were identified in two independent biological replicates according to the criteria for identification. A total of 489 proteins were screened as differentially expressed proteins. Among them, the expression of 227 DEPs was down‐regulated and 262 DEPs were up‐regulated. Detailed information of the DEPs is summarized in Table S3 with the ratio of iTRAQ reporter ion intensities. According to the statistics of gene ontology functional enrichment, most of the DEPs were related to the nucleus, nonmembrane‐bounded organelle, and cellular component organization or biogenesis (Figure 4a). According to pathway enrichment statistics, these DEPs participated in a variety of biological processes and the majority of them are involved in the biosynthesis of secondary metabolites, ribosome biogenesis, carbohydrate metabolism, and amino acid metabolism (Figure 4b).

### 3.5 Comparative analysis of RNA‐seq and iTRAQ dataset

**Figure 5:**
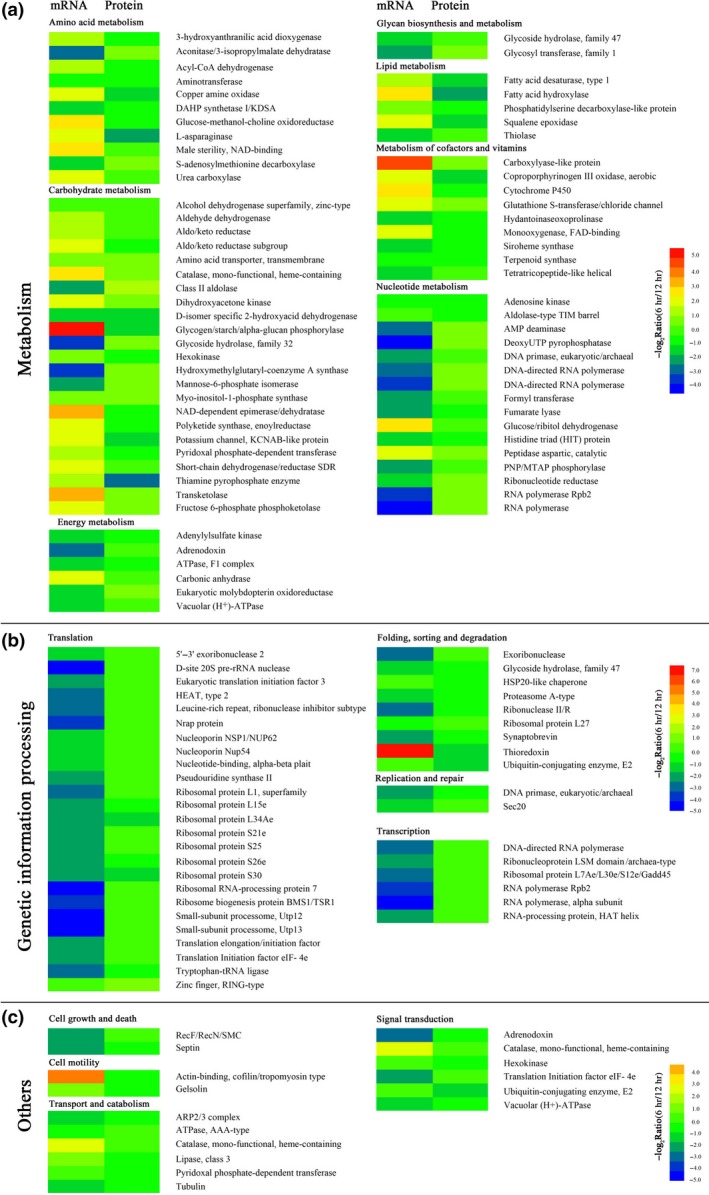
Expression changes of overlapping candidates involved in metabolism (a), genetic information processing (b) and others (c) from the iTRAQ and RNA‐seq datasets. Each row in the color heat map indicates a single protein. The annotation and involved metabolic pathway of each protein are listed. Blue to red in the color code indicates down‐ to up‐regulated expression of the candidates

**Figure 6:**
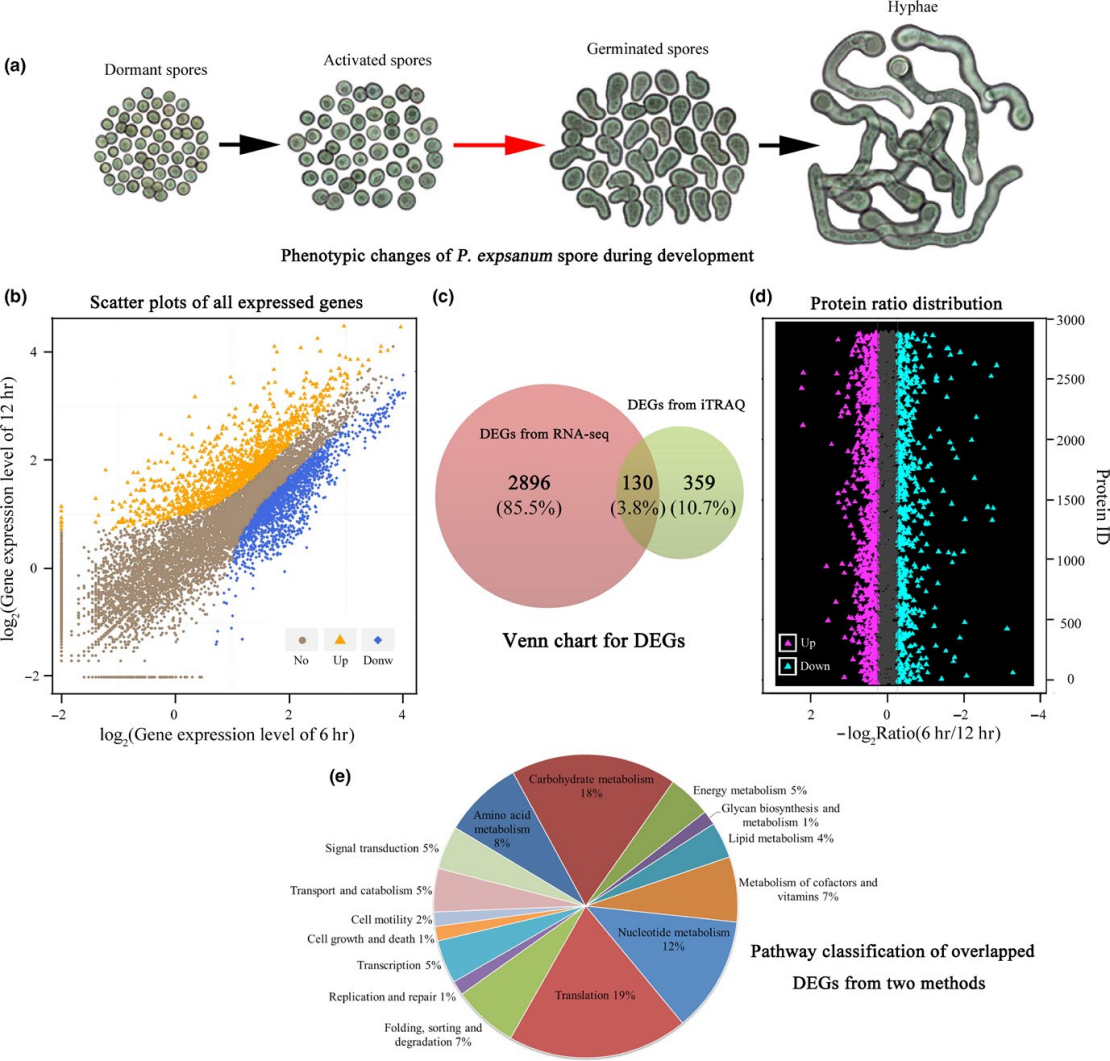
Global overview of the changes in phenotype, transcriptome and proteome of *P*. *expansum* spores during germination. (a) Phenotypic changes of *P*. *expansum* spores during development. The red arrow corresponds to the focused stage in this study; (b) Scatter plots of all expressed genes acquired from the RNA‐seq method during the spore germination stage. The *X*‐axis and *Y*‐axis show the log_2_ value of gene expression. Blue, orange, and brown dots correspond to down‐regulated, up‐regulated and nonregulated genes, respectively; (c) Venn diagram of the overlap of DEGs from RNA‐seq and iTRAQ datasets. (d) Protein ratio distribution acquired from the iTRAQ method during spore germination. The *X*‐axis presents the −log_2_ value of the protein ratio (6 hr/12 hr). Deep pink, azure, and gray dots correspond to up‐regulated, down‐regulated and nonregulated proteins, respectively. (e) Pathway classification of overlapped DEGs acquired from RNA‐seq and iTRAQ methods

Integrative analysis of transcriptomic and proteomic data may present complementary information that enables more informative conclusions to be drawn. After combination and simplification based on the homologies and biological function annotations and deleting the results with unknown functions, correlation analysis between iTRAQ and RNA‐seq datasets was performed. A total of 130 genes in the overlap were identified that were involved in amino acid metabolism (11), carbohydrate metabolism (23), energy metabolism (6), lipid metabolism (9), metabolism of cofactors and vitamins (9), nucleotide metabolism (16), translation (25), folding, sorting and degradation (9), transcription (6), transport and catabolism (6), signal transduction (6) or other pathways. Generally, the alterations of the expression of these genes showed a larger variation range at the level of transcription, and the expression trend of about half of the candidates was different between the transcriptional and translational levels (Figure 5 and Figure 6c and e). A poor correlation between the quantities of these two macromolecules indicated a complex regulatory mechanism controlling expression both at the RNA and the protein levels during spore germination of *P. expansum*. In fact, similar findings have previously been found in many other biological processes such as in humans, yeast, and plants (Hua et al., 2016).

## 4 DISCUSSION

*Penicillium expansum* is a destructive phytopathogen, capable of causing decay in deciduous fruits and vegetables during postharvest handling and storage. It also produces large amounts of secondary metabolites, especially patulin (Morales et al., 2010; Tannous et al., 2014). In this study, *P. expansum* was purified from naturally infected apple fruits and verified by morphological features and rDNA‐ITS analysis. The growth characteristics and pathogenicity of this pathogen were also determined (Figure 1, Figure 2 and Figure 6a). Then, changes in the transcriptome and proteome profiles at the germination stage were dissected utilizing RNA‐seq and iTRAQ approaches (Figure 6b and d). Correlation analysis between iTRAQ and RNA‐seq datasets was also performed (Figure 6c and e).

Fungal infection of plants is usually initiated by contact of the host with spores (conidia) and subsequently by germination. Germination is a highly coordinated, genetically programmed, and irreversible phenomenon that involves the initiation of biochemical activities with an increase in metabolism and an induction of morphological changes (Moir, 2006; Osherov & May, 2000). The first morphological change in spore germination is isotropic growth, also designated as swelling. Swelling is accompanied by water uptake, cell wall growth, changes in cellular composition, and a decrease in cytoplasmic microviscosity (Van Leeuwen, Van Doorn, Golovina, Stark, & Dijksterhuis, 2010). At the same time, swelling is accompanied by numerous metabolic activities including respiration and RNA and protein synthesis. Following swelling, cell wall deposition of polysaccharides such as chitin becomes polarized, and the extension of the fungal cell occurs at a restricted area at the tip of the cell. This results in elongation of a germ tube and the formation of a branching mycelium (Harris, 2006; Momany, 2002; Taheri‐Talesh et al., 2008; Van Leeuwen, Smant, De Boer, & Dijksterhuis, 2008).

A substantial amount of research on the germination process of bacterial or fungal spores has been published (Xiao, Francke, Abee, & Wells‐Bennik, 2011; Zhang, Garner, Setlow, & Yu, 2011; Gougouli & Koutsoumanis, 2012; Peleg & Normand, 2013; Olguín‐Araneda, Banawas, Sarker, & Paredes‐Sabja, 2015; Troiano, Zhang, Cowan, Yu, & Setlow, 2015). In particularly, gene expression profiles, either in the form of transcriptomes and/or proteomes, provide the means to explore and determine the underlying molecular and cellular processes of spore germination at an unprecedented depth and coverage. For example, using the Affymetrix GeneChip, Seong, Zhao, Xu, Güldener, and Kistler (2008) found that 185 genes were specifically expressed and 2,566 genes were significantly changed at different stages during spore germination of *Fusarium graminearum*. Most of these genes were involved in metabolism, carbohydrate utilization, and glycolysis and gluconeogenesis. Through DNA‐microarrays, Liu et al. (2007) provided a global view of an important system related to *Trichophyton rubrum* conidial germination via hierarchical clustering of 1,561 differentially expressed genes, and Bassi et al. (2016) found that 1,646 genes including the toxin‐coding genes *nheC*,* cytK* and *hblC* were differentially expressed and modulated during *Bacillus thuringiensis* spore germination and cell outgrowth in a vegetable‐based food model. Combining the 2‐DE‐based comparative proteomic approach and tandem electrospray ionization mass spectrometry (ESI‐MS/MS), Li, Lai, Qin, and Tian (2010) identified 34 proteins with significant changes in abundance when spore germination of *P. expansum* was inhibited by ambient pH and concluded that impairing the synthesis and folding of proteins was one of the main reasons. By utilizing a label‐free quantitative proteomics approach, Liu et al. (2016) found that 127 proteins were influenced during *Nosema bombycis* spore germination under extremely alkaline conditions.

However, these researchers focused on the molecular processes underlying spore germination at the single genome, transcriptional or protein level. In fact, molecular differences may be exhibited across multiple layers of gene regulation such as genomic variations, gene expression, protein translation and post‐translational modifications. Analyzing them separately may not be very informative. This necessitates the integrative analysis of such multiple layers of information to understand the interplay of the individual components.

When it comes to *P. expansum*, there are few reports about the changes in its gene expression during spore germination, since its genome was just completely sequenced in 2015 (Ballester et al., 2015; Li et al., 2015). In addition, integrating the information from transcriptomic and proteomic data to gain meaningful insights of fungal spore germination has rarely been reported. Therefore, two high‐throughput technologies of RNA‐seq and iTRAQ were used to explore global gene regulation during spore germination of *P. expansum* in this study. A total of 3,026 genes was found to be differently regulated, and 489 proteins showed variation in their abundance. Pathway classification was compared between DEGs from RNA‐seq and DEPs from iTRAQ analysis. Similar results were observed that the majority of DEGs or DEPs were involved in translation, transcription, and metabolism of carbohydrates, amino acids, lipids, energy, and nucleotides. They were absolutely required for spore metabolism and growth during germination. A total of 130 DEPs corresponded to gene sequences that were obtained by RNA‐seq, and this enabled a comparison of germination‐related differences in specific transcripts or cognate proteins. Therefore, by narrowing of the scope and reducing the number, the 130 overlapped DEPs could probably yield more precise and valuable information to explore the molecular mechanism of *P. expansum* germination.

Carbohydrates, technically hydrates of carbon and structurally polyhydroxy aldehydes and ketones, are typically stored as long polymers of glucose molecules with glycosidic bonds to provide structural support or energy storage. Carbohydrate metabolism denotes the various biochemical processes responsible for the formation, breakdown, and interconversion of carbohydrates. Carbohydrates serve as superior short‐term fuel for organisms because they are simpler to metabolize than lipids or amino acids. Typically, oligosaccharides and/or polysaccharides are cleaved into smaller monosaccharides and then are catabolized to produce energy, reducing powers, and components of other molecules. During germination with a relatively short time, up to 17.7% of the 130 DEPs were involved in carbohydrate metabolism such as glycolysis/gluconeogenesis (P3, P84, P193, P299, P330, and P367), butanoate metabolism (P68, P118, P412, P454, P464, and P461), glyoxylate and dicarboxylate metabolism (P72, P177, P396, and P477), fructose and mannose metabolism (P50, P115), starch and sucrose metabolism (P377), galactose metabolism (P64, P430), inositol phosphate metabolism (P227), and the pentose phosphate pathway (P126, P166). Most of these DEPs were up‐regulated in both mRNA and protein levels. This means that the metabolism of carbohydrates was necessary for the germination of *P. expansum* spores.

Proteins are large biomolecules, consisting of one or more long chains of amino acid residues. They are the primary participants within the cell that implement the changes specified by the information encoded in genes. With the exception of certain types of RNA, most other biological molecules are relatively inert elements upon which proteins act. In this study, 8.5% and 19.2% of DEPs were involved in amino acid metabolism and translation, respectively. With the exception of aminotransferase and DAHP synthetase, DEPs related to amino acid metabolism showed trends that changed differently at the mRNA and protein levels. In addition, the expression of all DEPs related to translation showed a decreasing trend at the mRNA level, but almost all of them showed an increasing trend at the protein level. Apparently, the translation system in *P. expansum* spores had been postponed. It was noteworthy that a large number of proteins related to the ribosome such as ribosome proteins and the processome, a ribosome assembly intermediate (Phipps, Charette, & Baserga, 2011), showed significant alterations of their expression levels. These changes significantly improved the output of translation initiation/elongation factors. A component protein of the proteasome whose function was to degrade unneeded or damaged proteins by proteolysis was decreased as well (Enenkel, 2014). Therefore, protein synthesis capacity during spore germination had increased and led to the subsequent changes in the biophysical or biochemical profiles of *P. expansum*.

Transcription is the first step of gene expression, in which a particular segment of DNA is copied into RNA, especially mRNA, by the enzyme RNA polymerase. In this study, almost all DEPs related to nucleotide metabolism and transcription were down‐regulated at the mRNA level and up‐regulated at the protein level. Among them, multiple types of RNA polymerases were notable, as they are necessary for constructing RNA chains using DNA genes as templates and are responsible for the synthesis of distinct subsets of RNA. These results indicated that genetic information processing had been activated and accelerated from dormancy to vegetative growth of *P. expansum* spores.

Energy metabolism is a set of metabolic reactions and processes to convert biochemical energy from nutrients into adenosine triphosphate (ATP) and release waste products. In this study, the expression levels of DEPs related to energy metabolism were all down‐regulated at both mRNA and protein levels. The possible reasons for this are delineated. Before germinating, the dormant spores need to shed their cell walls, degrade compatible sugars accompanied by a decrease in microviscosity of the cytoplasm, and reorganize the transcriptome including mRNA breakdown and the selected up‐regulation of different gene categories (Lamarre et al., 2008). Therefore, dormant spores need more energy than ones that have germinated. In addition, the DEPs related to transport, signal transduction, motility, and other molecular types of metabolism were recorded. It is reasonable that the conversion of dormant cells into vegetative cells requires active metabolism and cell division.

Overall, a large number of genes showed consistency between the transcript and protein levels, while some genes that exhibited inconsistency between these levels suggested that posttranscriptional regulation and modification serve important roles in the regulation of fungal germination. Additional research is needed to better understand the fact that the turnover and stability of mRNA levels are important for the translation of mRNA into proteins. For example, the down‐regulation of expression at the transcriptional level combined with the up‐regulation of expression at the protein level of some DEGs implied that mRNA had already begun to be degraded. Conversely, there may be regulation of translational pathways or increased protein degradation leading to apparently enhanced transcription. In summary, the transcriptomic and proteomic data in this study not only highlighted a set of genes and proteins that were important in deciphering the molecular processes of the germination of *P. expansum* but also provided the basic knowledge for the development of effective control methods and adequate environmental conditions.

## CONFLICT OF INTEREST

None declared.
