# Supplementary figures and images for: Identification of differentially expressed genes involved in spore germination of Penicillium expansum by comparative transcriptome and proteome approaches
Source: Microbiologyopen. 2017 Dec 5;7(3):e00562. doi: 10.1002/mbo3.562 (PMC6011939; doi:10.1002/mbo3.562)

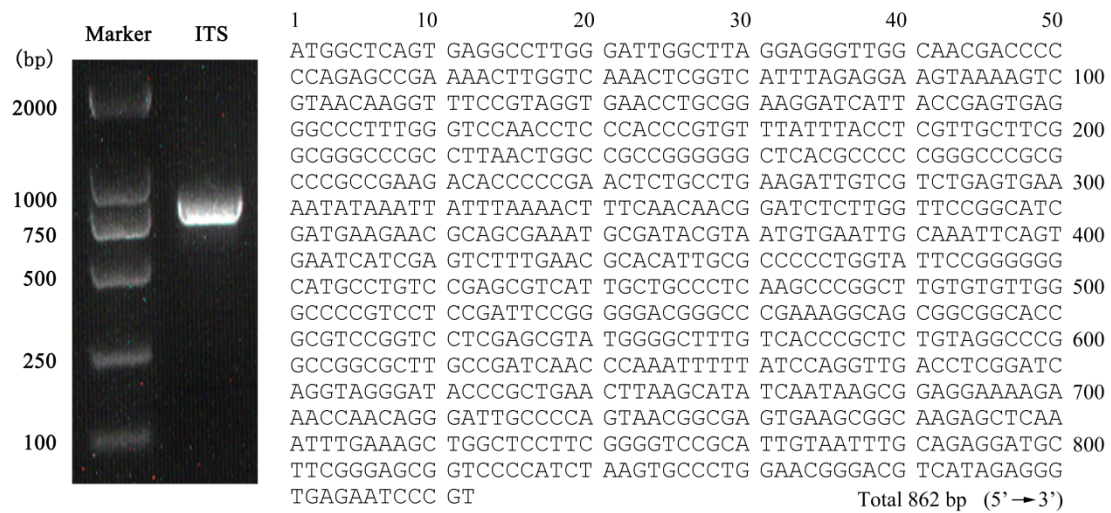

**Figure S1.**

Supplement: Supplementary file 1 [file MBO3-7-e00562-s001.pdf]
